# Supplementary material for: A Comparative Study on Cognitive Assessment in Cerebellar and Supratentorial Stroke
Source: Brain Sci. 2024 Jul 3;14(7):676. doi: 10.3390/brainsci14070676 (PMC11274804; doi:10.3390/brainsci14070676)
Supplement: Supplementary file 1 [file brainsci-14-00676-s001.zip › brainsci-3034898-supplementary.pdf]

**Supplementary Material**

**Table S1. Introduction to the evaluation of the cognitive function.**

| Number | The name of the scale | Introduction of the scale | sensitivity and specificity of the scale |
|---|---|---|---|
| 1 | Mini-Mental State Examination (MMSE) | The MMSE assesses different cognitive domains: orientation to time and place, registration and recall, attention and calculation, language abilities, and basic motor skills. The test consists of 11 items with a maximum score of 30 points. | A score of 23 or lower indicates cognitive impairment, with the pooled sensitivity of 88.3% and specificity of 86.2% for dementia [1, 2]. |
| 2 | Montreal Cognitive Assessment (MoCA) | The MoCA is a widely used screening tool for cognitive impairment, designed to detect mild cognitive dysfunction [3]. It assesses various cognitive domains, including visuospatial abilities, memory, attention, language, abstraction, and executive function. The MoCA is typically administered in about 10 minutes and scored out of 30. | A score of 26 or lower indicates cognitive impairment [3]. A study conducted in China found that MoCA have a sensitivity of 89.0% and specificity of 80.0% in vascular dementia patients [4]. |
| 3 | Addenbrooke's Cognitive Examination III (ACE-III) | The ACE-III is a comprehensive cognitive assessment tool that evaluates attention, memory, verbal fluency, language, and visuospatial abilities, with scores ranging from 0 to 100 [5]. | Cognitive impairment was defined as an ACE-III score below 87, which has been validated in the Chinese population, with the sensitivity of 75.0% and specificity of 89.0% [6]. |
| 4 | Cerebellar Cognitive Affective Syndrome scale (CCAS-s) | The CCAS-s was used to assess Cerebellar Cognitive Affective Syndrome[7]. The scale contains 10 items and each item has a designated cut-off score to determine pass or fail. CCAS is considered possible if one test is failed, probable if two tests are failed, and definite if three or more tests are failed. | Based on the definition, the sensitivity of the Chinese version of CCAS-s to identify possible/probable/definite CCAS was 80.0%/53.3%/40.0% and the specificity was 40.0%/80.0/96.7% [8]. |
| 5 | Trail Making Test (TMT) | The TMT is a neuropsychological assessment of visual attention and task switching. It consists of two parts, A and B, in which the individual is instructed to connect a set of 25 dots as | The sensitivity and specificity of TMT B were 28.0% and 94.0% with a cutoff of 26, whereas for TMT A, they |

| | | | |
|---|---|---|---|
| | | quickly as possible while still maintaining accuracy [9]. | were 44.0% and 91.0% with a cutoff of 30 [10]. |
| 6 | Digit Span Test (DST) | The forward and backward DST are widely used measures of working memory capacity and have been used in various populations, including individuals with vascular dementia [11, 12]. The Digit Span Test is a subtest of the Wechsler Adult Intelligence Scale and is a simple and effective way to assess working memory. The Digit Span Forward (DSF) process involves reading a sequence of digits at a rate of one digit per second, with the subject asked to repeat it immediately in sequential form. On the other hand, the Digit Span Backward (DSB) process entails reading a sequence of digits at a rate of one digit per second, and the subject is required to repeat it immediately in reverse order. | DSF demonstrated a sensitivity of 46.0% and specificity of 77.0% at the optimal cutoff of 8. In comparison, DSB exhibited a sensitivity of 77.0% and specificity of 78.0% at the optimal cutoff of 3 [13]. |
| 7 | Rey Complex Figure (RCF) test | The RCF test is a widely used neuropsychological assessment tool that involves copying, immediate recall, and delayed recall of a complex figure [14]. The RCF test is a sensitive measure of visuospatial and constructional abilities, and visual memory. The test involves the presentation of a two-dimensional figure that the examinee must copy (i.e., the Copy Trial) and reproduce from memory after 3-min and 30-min delay periods. | The sensitivity of this scale for detecting mild cognitive impairment is among 78.0-85.0%, while the specificity is among 50.0-90.0% [15]. |
| 8 | Boston Naming Test (BNT) | The BNT measures an individual's ability to name objects or items presented to them [16]. Subjects were asked to spontaneously name 30 pictures and were considered to be in error if they answered incorrectly or could not answer within 20 seconds. | The sensitivity of this scale for detecting mild cognitive impairment is 74.5% with a cutoff value of 25, while the specificity is 54.5%. For detecting dementia, the sensitivity of this scale is |

| | | | |
|---|---|---|---|
| | | The indicator of observation is the number of correct naming. | 63.5% with a cutoff value of 22, while the specificity is 82.2% [17]. |
| 9 | Rey Auditory Verbal Learning Test (RAVLT) | Rey Auditory Verbal Learning Test (RAVLT) is a widely used neuropsychological assessment tool that evaluates an individual's ability to encode, consolidate, and retrieve verbal information [18]. This test involves the presentation of a list of 12 unrelated words multiple times, followed by a delayed recall and recognition phase. | The scale showed strong classification accuracy in detecting mild memory impairment with a cut-score of 13, achieving a sensitivity of 82.0% and a specificity of 89.0%. Although accuracy decreased in individuals with severe memory impairment, it still demonstrated significance with an alternative cut-score of 11, achieving a sensitivity of 37.0% and a specificity of 88.0% [19]. |
| 10 | Verbal fluency test (VFT) | This test provides valuable information about language and executive functioning abilities. There are two main types of VFT: Phonemic Fluency and Semantic Fluency. In Phonemic fluency, participants are asked to generate as many words as possible that begin with a specific letter: 车 (The word means 'car' in English) within one minute. Semantic fluency involves generating words that belong to a specific category (animals) within one minute [20, 21]. | Phonemic Fluency showed a sensitivity of 80.0% and specificity of 57.0% at the optimal cutoff of 7. The sensitivity and specificity of Semantic Fluency were 83.0% and 42.0% at the optimal cutoff of 14 [22]. |
| 11 | the Stroop test | The Stroop Test examines an individual's cognitive processing speed and their ability to inhibit automatic responses, it also assess cognitive functioning and detect impairments in executive functions, such as attention, cognitive flexibility, and response inhibition [23]. In the Stroop Test, individuals are presented with a series of color words (e.g., red, blue, green) | The test had a sensitivity of 79.0% and a specificity of 89.0% in differentiating between AD patients and healthy controls [24]. |

| | | | |
|---|---|---|---|
| | | that are printed in incongruent colors (e.g., the word "red" printed in blue ink). The task requires the individual to name the color of the ink while ignoring the written word. The interference effect observed when individuals struggle to inhibit the automatic response of reading the word is used to evaluate cognitive control and attentional processing. | |

**Table S2. Demographic and clinical characteristics of patients with cerebellar infarction, patients with frontal infarction, and healthy controls.**

| | Cerebellar infarction patients n=50 | Frontal infarction patients n=38 | Healthy controls n=39 | *P* value |
|---|---|---|---|---|
| Age (y) | 53.1±12.5 | 49.8±11.7 | 50.8±7.8 | 0.31 |
| Male, n (%) | 39 (78.0) | 27(71.1) | 30 (76.9) | 0.73 |
| Education (y) | 11.1±3.0 | 11.6±3.1 | 11.0±2.0 | 0.74 |
| BMI (kg/m2) | 23.4±0.4 | 22.9±0.3 | 22.7±0.4 | 0.33 |
| Smoking (n, %) | 15 (30.0) | 20 (52.6) | 12 (30.8) | 0.18 |
| Drinking (n, %) | 16 (32.0) | 12 (31.6) | 10 (25.6) | 0.65 |
| Medical History (n, %) | | | | |
| Hypertension | 34 (68.0) | 29 (76.3) | 24 (61.5) | 0.38 |
| Diabetes | 18 (36.0) | 11 (28.9) | 12 (30.8) | 0.36 |
| Dyslipidemia | 11 (22.0) | 11 (28.9) | 15 (38.5) | 0.24 |
| Disease duration (d) | 8.7±4.9 | 7.3±4.5 | - | 0.05 |
| Lesion volume ($cm^3$) | 17.0±8.1 | 16.9±7.8 | - | 0.90 |
| NIHSS score | 1.1±0.9 | 1.1±1.3 | - | 0.52 |
| ICARS score | 7.5±6.9 | - | - | - |
| BBA score | 11.0±1.4 | - | - | - |
| SAS score | 36.3±5.5 | 35.7±6.6 | 31.6±6.0 | 0.002[a, b] |
| SDS score | 35.7±6.7 | 35.6±6.4 | 35.6±5.8 | 0.96 |

[a]: patients with cerebellar infarction were significantly different from healthy controls ($P < 0.05$); [b]: patients with supratentorial infarction were significantly different from healthy controls ($P < 0.05$). Abbreviations: BMI: Body mass index; NIHSS: National Institute of Health stroke scale; ICARS: International Cooperative Ataxia Rating Scale; BBA: Brunel balance assessment; SAS: self-rating anxiety scale; SDS: self-rating depression scale.

**Table S3. ROC analyses for MMSE, MoCA, ACE-III, CCAS-s total score and CCAS-s failed items number to differentiate patients with cerebellar infarction, patients with supratentorial infarction, and patients with frontal infarction from cognitively normal controls**

| Groups | AUC | 95% Confidence interval | cutoff value | Sensitivity (%) | Specificity (%) |
|---|---|---|---|---|---|
| Patients with cerebellar infarction vs. Healthy controls | | | | | |
| MMSE | 0.50 | 0.38-0.62 | | - | |
| MoCA | 0.56 | 0.44-0.68 | | - | |
| ACE-III | 0.72*** | 0.62-0.83 | 88 | 100.0 | 40.0 |
| CCAS-s | 0.67** | 0.56-0.78 | 91 | 94.9 | 48.0 |
| Number of CCAS-s failed tests | 0.69*** | 0.59-0.80 | 2 | 44.0 | 87.2 |
| Patients with supratentorial infarction vs. Healthy controls | | | | | |
| MMSE | 0.62* | 0.51-0.72 | 25 | 100 | 38.3 |
| MoCA | 0.70*** | 0.60-0.80 | 25 | 100 | 38.3 |
| ACE-III | 0.80*** | 0.72-0.89 | 88 | 100 | 53.3 |
| CCAS-s | 0.79*** | 0.70-0.88 | 91 | 94.9 | 60.0 |
| Number of CCAS-s failed tests | 0.82*** | 0.74-0.90 | 2 | 71.7 | 87.2 |
| Patients with frontal infarction vs. Healthy controls | | | | | |
| MMSE | 0.57 | 0.43-0.70 | | - | |
| MoCA | 0.68** | 0.56-0.81 | 25 | 100 | 42.1 |
| ACE-III | 0.74*** | 0.62-0.85 | 88 | 100 | 52.6 |
| CCAS-s | 0.76*** | 0.65-0.87 | 91 | 94.9 | 57.9 |
| Number of CCAS-s failed tests | 0.79*** | 0.69-0.89 | 2 | 68.4 | 87.2 |

Abbreviations: ROC: receiver operating characteristic; AUC: Area Under Curve; MMSE: Mini-Mental State Examination; MoCA: Montreal Cognitive Assessment; ACE-III: Addenbrooke's Cognitive Examination III; CCAS-s: Cerebellar Cognitive Affective Syndrome scale. *$p < 0.05$; ** $p < 0.01$; ***$p < 0.001$.

**Table S4. Performance on neuropsychological tests of patients with cerebellar infarction, patients with supratentorial infarction, and healthy controls**

| Neuropsychological tests | Cerebellar infarction patients n=50 | Supratentorial infarction patients n=60 | Healthy controls n=39 | P value |
|---|---|---|---|---|
| Attention | | | | |
| TMT-A (s) | 42.7±20.0 | 44.0±13.0 | 35.6±12.2 | 0.003[b] |
| Forward digit span | 8.6±1.2 | 7.6±1.0 | 9.4±0.9 | <0.001[a, b, c] |
| Visuospatial function | | | | |
| RCF copy | 34.9±2.1 | 34.9±2.0 | 34.7±1.9 | 0.08 |
| Language function | | | | |
| BOSTON naming | 26.4±2.1 | 26.7±2.0 | 26.5±1.7 | 0.67 |
| Episodic memory | | | | |
| RCF (%) | 56.6±17.8 | 58.3±17.3 | 62.3±14.2 | 0.26 |
| RAVLT (%) | 80.0±18.1 | 72.9±17.3 | 81.7±13.6 | 0.01[b] |
| Executive function | | | | |
| TMT B-A (s) | 40.3±20.7 | 38.4±14.3 | 39.3±16.4 | 0.96 |
| Phonemic fluency | 9.9±3.1 | 10.1±3.4 | 11.6±2.2 | 0.01[a,b] |
| Semantic fluency | 18.3±3.1 | 18.1±3.4 | 21.1±2.2 | <0.001[a,b] |
| SIE-time (s) | 36.8±17.9 | 36.0±16.7 | 32.5±12.2 | 0.49 |
| SIE-correct | 1.8±2.1 | 2.0±2.3 | 0.8±1.1 | 0.01[b] |

a: patients with cerebellar infarction (CI) were significantly different from healthy controls (HC) ($p<0.05$); b: patients with supratentorial infarction (SI) were significantly different from healthy controls ($p<0.05$); c: patients with supratentorial infarction were significantly different from patients with cerebellar infarction ($p<0.05$). Missing data: TMT-A: 4CI, 2SI; Forward digit span: 2CI; RCF copy: 2CI; BOSTON naming: 2CI; RCF: 7CI, 7SI; RAVLT: 1HC, 9CI, 4SI; TMT B-A: 5CI, 2SI; SIE-time: 4CI, 6SI; SIE-correct:4CI, 6SI. Abbreviations: TMT: Trail Making Test; RCF: Rey Complex Figure; RAVLT: Rey Auditory Verbal Learning test; SIE: Stroop Interference Effect.

**Table S5. Performance on neuropsychological tests of patients with cerebellar infarction, patients with frontal infarction, and healthy controls**

| Neuropsychological tests | Cerebellar infarction patients n=50 | Frontal infarction patients n=38 | Healthy controls n=39 | *P* value |
|---|---|---|---|---|
| Global cognitive function | | | | |
| MMSE | 27.7±1.6 | 26.2±3.7 | 27.7±1.3 | 0.45 |
| MoCA | 27.5±1.5 | 26.3±2.3 | 27.9±1.3 | 0.01[b] |
| ACE-III | 89.8±4.7 | 89.4±4.8 | 93.3±2.9 | <0.001[a, b] |
| CCAS-s | 93.1±10.1 | 90.4±9.1 | 99.0±6.1 | <0.001[a, b] |
| Number of CCAS-s failed tests | 1.8±1.7 | 2.03±1.3 | 0.7±0.8 | <0.001[a, b] |
| Attention | | | | |
| TMT-A (s) | 42.7±20.0 | 43.2±12.3 | 35.6±12.2 | 0.02[b] |
| Forward digit span | 8.6±1.2 | 7.6±1.0 | 9.4±0.9 | <0.001[a, b, c] |
| Visuospatial function | | | | |
| RCF copy | 34.9±2.1 | 34.9±2.0 | 34.7±1.9 | 0.09 |
| Language function | | | | |
| BOSTON naming | 26.4±2.1 | 26.8±2.1 | 26.5±1.7 | 0.46 |
| Episodic memory | | | | |
| RCF (%) | 56.6±17.8 | 60.0±18.4 | 62.3±14.2 | 0.26 |
| RAVLT (%) | 80.0±18.1 | 79.5±17.5 | 81.7±13.6 | 0.77 |
| Executive function | | | | |
| TMT B-A (s) | 40.3±20.7 | 38.7±14.8 | 39.3±16.4 | 0.98 |
| Phonemic fluency | 9.9±3.1 | 9.9±3.6 | 11.6±2.2 | 0.007[a, b] |
| Semantic fluency | 18.3±3.1 | 17.8±3.4 | 21.1±2.2 | <0.001[a, b] |
| SIE-time (s) | 36.8±17.9 | 36.3±18.2 | 32.5±12.2 | 0.49 |
| SIE-correct | 1.8±2.1 | 1.7±2.4 | 0.8±1.1 | 0.06 |

a: patients with cerebellar infarction (CI) were significantly different from healthy controls (HC) (p<0.05); b: patients with frontal infarction (FI) were significantly different from healthy controls (p<0.05); c: patients with supratentorial infarction were significantly different from patients with cerebellar infarction (p<0.05). Missing data: TMT-A: 4CI, 1FI; Forward digit span: 2CI; RCF copy: 2CI; BOSTON naming: 2CI; RCF: 7CI, 7FI; RAVLT: 1HC, 9CI, 4FI; TMT B-A: 5CI, 1FI; SIE-time: 4CI, 5FI; SIE-correct:4CI, 5FI. Abbreviations: MMSE: Mini-Mental State Examination; MoCA: Montreal Cognitive Assessment; ACE-III: Addenbrooke's Cognitive Examination III; CCAS-s: Cerebellar Cognitive Affective Syndrome scale; TMT: Trail Making Test; RCF: Rey Complex Figure; RAVLT: Rey Auditory Verbal Learning test; SIE: Stroop Interference Effect.

**Reference**


[1] Katzman R, Zhang MY, Ouang Ya Q, Wang ZY, Liu WT, Yu E, *et al.* (1988). A Chinese version of the Mini-Mental State Examination; impact of illiteracy in a Shanghai dementia survey. J Clin Epidemiol, 41:971-978.

[2] Arevalo-Rodriguez I, Smailagic N, Roqué IFM, Ciapponi A, Sanchez-Perez E, Giannakou A, *et al.* (2015). Mini-Mental State Examination (MMSE) for the detection of Alzheimer's disease and other dementias in people with mild cognitive impairment (MCI). Cochrane Database Syst Rev, 2015:Cd010783.

[3] Hobson J (2015). The Montreal Cognitive Assessment (MoCA). Occup Med (Lond), 65:764-765.

[4] Dong Y, Sharma VK, Chan BP, Venketasubramanian N, Teoh HL, Seet RC, *et al.* (2010). The Montreal Cognitive Assessment (MoCA) is superior to the Mini-Mental State Examination (MMSE) for the detection of vascular cognitive impairment after acute stroke. J Neurol Sci, 299:15-18.

[5] Hsieh S, Schubert S, Hoon C, Mioshi E, Hodges JR (2013). Validation of the Addenbrooke's Cognitive Examination III in frontotemporal dementia and Alzheimer's disease. Dement Geriatr Cogn Disord, 36:242-250.

[6] Li X, Yang L, Yin J, Yu N, Ye F (2019). Validation Study of the Chinese Version of Addenbrooke's Cognitive Examination III for Diagnosing Mild Cognitive Impairment and Mild Dementia. J Clin Neurol, 15:313-320.

[7] Hoche F, Guell X, Vangel M, Sherman J, Schmahmann J (2018). The cerebellar cognitive affective/Schmahmann syndrome scale. Brain : a journal of neurology, 141:248-270.

[8] Liu Q, Liu C, Nan S, Wang P, Zhang Y, Chen Y Recognition of Cognitive Dysfunction in Cerebellar Infarction: Validation of the Chinese Cerebellar Cognitive Affective Syndrome Scale, 13 June 2023, PREPRINT (Version 1) available at Research Square [https://doi.org/10.21203/rs.3.rs-3054967/v1].

[9] Tombaugh T (2004). Trail Making Test A and B: normative data stratified by age and education. Arch Clin Neuropsychol, 19:203-214.

[10] Peak AM, Marceaux JC, Chicota-Carroll C, Soble JR (2024). Cross-validation of the Trail Making Test as a non-memory-based embedded performance validity test among veterans with and without cognitive impairment. J Clin Exp Neuropsychol, 46:16-24.

[11] Wechsler D. 1997. *WAIS-III administration and scoring manual.*: San Antonio, TX: Psychological Corporation.

[12] Wechsler D. 1998. *Wechsler Memory Scale—third edition (WMS-III).* London, UK: Psychological Corp.

[13] Leung JL, Lee GT, Lam YH, Chan RC, Wu JY (2011). The use of the Digit Span Test in screening for cognitive impairment in acute medical inpatients. Int Psychogeriatr, 23:1569-1574.

[14] Rey A (1941). The psychological examination in cases of traumatic encepho- lopathy. Problems [in French]. Arch Psychol 28:215-218.

[15] Simfukwe C, An SS, Youn YC (2021). Comparison of RCF Scoring System to Clinical Decision for the Rey Complex Figure Using Machine-Learning Algorithm. Dement Neurocogn Disord, 20:70-79.

[16] Kaplan E, Goodglass H, Weintraub S. 2001. *Boston Naming Test*: Austin Texas: Pro-ed.

[17] Li D, Yu YY, Hu N, Zhang M, Liu L, Fan LM, *et al.* (2022). A Color-Picture Version of Boston Naming Test Outperformed the Black-and-White Version in Discriminating Amnestic Mild Cognitive Impairment and Mild Alzheimer's Disease. Front Neurol, 13:884460.

[18] Rey A. 1958. *The clinical examination in psychology [in Frenc].* Paris, France: Presses Universitaires de France.

[19] Soble JR, Sharp DW, Carter DA, Jennette KJ, Resch ZJ, Ovsiew GP, *et al.* (2021). Cross-validation of a forced-choice validity indicator to enhance the clinical utility of the Rey Auditory Verbal Learning Test. Psychol Assess, 33:568-573.

[20] Spreen O, Strauss E. 1998. *A compendium of neuropsychological tests: administration, norms, and commentary.*: Oxford, UK: Oxford University Press.

[21] Tombaugh T, Kozak J, Rees L (1999). Normative data stratified by age and education for two measures of verbal fluency: FAS and animal naming. Arch Clin Neuropsychol, 14:167-177.

[22] Muangpaisan W, Intalapaporn S, Assantachai P (2010). Digit span and verbal fluency tests in patients with mild cognitive impairment and normal subjects in Thai-community. J Med Assoc Thai, 93:224-230.

[23] Stroop J (1935). Studies of interference in serial verbal reactions. Journal of Experimental Psychology, 18:643-662.

[24] Snyder HR (2013). Major depressive disorder is associated with broad impairments on neuropsychological measures of executive function: a meta-analysis and review. Psychol Bull, 139:81-132.
